# Supplementary material for: Clinical anastomosis leakage and associated factors among patients who had intestinal anastomosis in northwest referral hospitals, Ethiopia
Source: PLoS One. 2022 Dec 30;17(12):e0275536. doi: 10.1371/journal.pone.0275536 (PMC9803163; doi:10.1371/journal.pone.0275536)
Supplement: Attachment — Submitted filename: Review comments.docx [file pone.0275536.s001.docx]

Review comments

Manuscript ID PONE-D-22-13405

**Title**: Clinical anastomosis leakage and associated factors among patients who had intestinal anastomosis in northwest Amhara regional state referral hospitals, Ethiopia, 2020

Thank you for inviting me to review this interesting research. I read and review the research with great interest. The author tried to describe the Clinical anastomosis leakage and associated factors among patients who had intestinal anastomosis in southwest, Ethiopia. My comments are listed below;

- This manuscript needs to be reviewed for typographical, syntax, spelling, and grammatical errors.
- Please rewrite your title as ‘Clinical anastomosis leakage and associated factors among patients who had intestinal anastomosis in northwest referral hospitals, Ethiopia’. The details are better explained in the methods section.

**Introduction**

Your introduction section is well written but,

- The authors stated more about scientific/known facts
- didn’t answer well the research question, it needs further elaboration to full fill the scientific merits.
- you do not explain the global and national efforts to tackle the problem, gaps in previous scholars that your study is trying to fill in.

The last paragraph in your introduction said ‘Since there is no study, which shows both the prevalence and factors associated with anastomotic leak in our study setting.’ What does it mean? The sentence is not completed. Moreover, why do you need to stick anastomotic leak in your study setting only? Does every specific setting need research? what is the advancement of new knowledge by this study from <http://dx.doi.org/10.4314/ejhs.v29i6.14> ?

**Methods**

- Please state the total hospitals that perform major operations/surgery in the study setting.
- ‘The data was extracted from March 1 to June,2020……’ please correct English as data were…

**Sample size determination, Sampling procedure, and study variables**

Show the calculation for the sample size.

The sampling procedure is not clear, the readers left confused.

- First, how three hospitals were selected?
- It is not clear on how many total eligible cases (patient cards/records) were entertained per each hospital?
- The proportion of sample contributed per each hospital?
- I suggest using a diagram for more information regarding
- I have a doubt regarding some explanatory variables you really accessed from secondary data/record patient occupation. Please comment on this. And/or acknowledge your limitations.
- How do you measure the utilization of alcohol and smoking? The dose and frequency of usage matter? In a lifetime, per day weekly, on holydays only…; also amount?

**Data processing and analysis**

What does it mean you have used SPSS v. 24 in your abstract section and v.25 in the main text?

**Discussion**

I suggest that discussion section start with a paragraph highlighting the most relevant accomplishments of the research.

This section can be improved as follows.

a. Main findings in the first paragraph

b. Comparison with existing literature

c. Strengths and Weaknesses of the study should be clearly highlighted

d. Conclusion

**Generally**,

-please, read the complete manuscript and correct typos.

-Please ensure that your manuscript meets PLOS ONE's style requirements, including those for file naming. The PLOS ONE style templates.

Best wishes!
