# Supplementary material for: Clinical anastomosis leakage and associated factors among patients who had intestinal anastomosis in northwest referral hospitals, Ethiopia
Source: PLoS One. 2022 Dec 30;17(12):e0275536. doi: 10.1371/journal.pone.0275536 (PMC9803163; doi:10.1371/journal.pone.0275536)
Supplement: Attachment — Submitted filename: Response to to reviews.docx [file pone.0275536.s002.docx]

**Point by point response to Reviewer’s response**

**Response to Reviewer #1**

| **S.No** | **Issues raised** | **Reponses** |
| --- | --- | --- |
| 1. | The authors did not analysis the nutrition status, intra-abdominal infection, or other modifiable risk factors-such as operative time, use of drainage tube etc. So, they cannot give the readers more important things to prevent anastomosis leakage. Most of their patients were large bowel related disease (50.9%), and majority of operation was on emergency basis (57.4%). in this situation, most of patients may not have bowel preparation. And their use of drainage tube rate was only 4.9%, most of them (95.1%) did not have adequate drainage. | - Stated in the limitation as “This study did not assess association of this variables with clinical anastomosis leakage. But, operative time and use of drainage tube have failed to pass the chi-square test assumption, and presented descriptively. |
| 2. | In abstract, they use “SPSS version 24”, but in the text, it becames “SPSS 25”. | We used SPSS version -25 for analysis, and modified as SPSS version -25 |
| 3. | In table 2, the percentage of ASA class I&II and class III&IV seems reverse. | Modified as per the recommendation |
| 4. | In table 5, the authors did not analysis the effect of drainage tube. | We did not include the effect of drainage tube as it fails to pass the chi-square test assumption. |
| 5. | Repeat sentence in Methods and Materials—Population—(All surgical……in the study) | Modified |

**Response to Reviewer#2**

| **S.No** | **Manuscript section** | **Issues raised** | **Reponses** |
| --- | --- | --- | --- |
| 6. | General | This manuscript needs to be reviewed for typographical, syntax, spelling, and grammatical errors. | Modified |
| 7. | Title | Rewrite your title as ‘Clinical anastomosis leakage and associated factors among patients who had intestinal anastomosis in northwest referral hospitals, Ethiopia’. | Modified as per the recommendation |
| 8. | Introduction | The authors stated more about scientific/known facts | we tried and modified |
|  |  | Didn’t answer well the research question, it needs further elaboration to full fill the scientific merits. | we tried and modified |
|  |  | You do not explain the global and national efforts to tackle the problem, gaps in previous scholars that your study is trying to fill in. | WHO guidelines for safe-surgery identify potential standards for enhancements in four areas: safe surgical teams, safe anesthesia, prevention of surgical site infection, and measurement of surgical services |
|  |  | The last paragraph in your introduction said ‘Since there is no study, which shows both the prevalence and factors associated with anastomotic leak in our study setting.’ What does it mean? The sentence is not completed. Moreover, why do you need to stick anastomotic leak in your study setting only? Does every specific setting need research? what is the advancement of new knowledge by this study from <http://dx.doi.org/10.4314/ejhs.v29i6.14> ? | - The last paragraph is modified as “There is limited study on magnitude and factors associated with anastomotic leak in Ethiopia in the study area in particular. Before forwarding suggestions, knowing the burden and the factors that associate with anastomotic leakage plays vital role in preventing for its occurrence.” - As we stated in the statement of problem there is limited evidence on anastomotic leakage in Ethiopia. There is a need to have more evidences to support the actions with evidences. But, every specific setting does not need research. |
| 9. | **Methods** | Please state the total hospitals that perform major operations/surgery in the study setting. | - Modified as per the recommendation, and highlighted in the tracked document. |
|  |  | The data was extracted from March 1 to June, 2020……’ please correct English as data were… | - Modified as per the recommendation, and highlighted in the tracked document. |
| 10. | **Sample size determination, Sampling procedure, and study variables** | Show the calculation for the sample size.  The sampling procedure is not clear, the readers left confused.   - First, how three hospitals were selected? - It is not clear on how many total eligible cases (patient cards/records) were entertained per each hospital? - The proportion of sample contributed per each hospital? - I suggest using a diagram for more information regarding - I have a doubt regarding some explanatory variables you really accessed from secondary data/record patient occupation. Please comment on this. And/or acknowledge your limitations. - How do you measure the utilization of alcohol and smoking? The dose and frequency of usage matter? In a lifetime, per day weekly, on holydays only…; also amount? | - Modified as per the recommendation, and highlighted in the tracked document. - The frequency and dose of the utilization of alcohol and smoking were not measured. Rather, we took what was recorded in the history of smoking or alcohol in the patient chart. We stated it in the limitation of the study. |
| 11. | **Data processing and analysis** | What does it mean you have used SPSS v. 24 in your abstract section and v.25 in the main text? | - Modified as per the recommendation, and highlighted in the tracked document. |
| 12. | **Discussion** | I suggest that discussion section start with a paragraph highlighting the most relevant accomplishments of the research.  This section can be improved as follows.  a. Main findings in the first paragraph  b. Comparison with existing literature  c. Strengths and Weaknesses of the study should be clearly highlighted  d. Conclusion | - Modified as per the recommendation, and highlighted in the tracked document. |
