# Supplementary material for: Clinical anastomosis leakage and associated factors among patients who had intestinal anastomosis in northwest referral hospitals, Ethiopia
Source: PLoS One. 2022 Dec 30;17(12):e0275536. doi: 10.1371/journal.pone.0275536 (PMC9803163; doi:10.1371/journal.pone.0275536)
Supplement: Attachment — Submitted filename: Response to to reviews.docx [file pone.0275536.s003.docx]

**Point by point response to editors and Reviewers’ comments**

**Response to editors #1**

| **Section** | | **Issues raised** | **Reponses** |
| --- | --- | --- | --- |
| Journal Requirements | References issues | Please review your reference list to ensure that it is complete and correct. | - We have checked the references , and it is complete and correct |
|  | Financial disclosure | If you would like to make changes to your financial disclosure, please include your updated statement in your cover letter. | No change is made in the financial disclosure |
|  | Figure formatting | Guidelines for resubmitting your figure files are available below the reviewer comments at the end of this letter. | PACE is used to format the figures |

**Response to Reviewer#1**

| **S.No** | **Manuscript section** | **Issues raised** | **Reponses** |
| --- | --- | --- | --- |
| 1. | Introduction and result | The most location of intestinal anastomosis was in large intestine (50.9%), and as we know, rates of anastomotic leak vary based on the type of anastomosis with entero-enteric having the lowest leak rate (1–2%) and colorectal/coloanal having the highest (4–26%). Why did the authors do not place a drainage tube for them, only 4.9%? | We were unable to do that because we used secondary data. We took what was recorded in the patients’ record. We stated this in the limitation section. |
